# Expression level, cellular compartment and metabolic network position all influence the average selective constraint on mammalian enzymes

**DOI:** 10.1186/1471-2148-11-89

**Published:** 2011-04-06

**Authors:** Corey M Hudson, Gavin C Conant

**Affiliations:** 1 Informatics Institute, University of Missouri, Columbia, MO, USA; 2 Division of Animal Sciences, University of Missouri, Columbia, MO, USA

## Abstract

**Background:**

A gene's position in regulatory, protein interaction or metabolic networks can be predictive of the strength of purifying selection acting on it, but these relationships are neither universal nor invariably strong. Following work in bacteria, fungi and invertebrate animals, we explore the relationship between selective constraint and metabolic function in mammals.

**Results:**

We measure the association between selective constraint, estimated by the ratio of nonsynonymous (K_a_) to synonymous (K_s_) substitutions, and several, primarily metabolic, measures of gene function. We find significant differences between the selective constraints acting on enzyme-coding genes from different cellular compartments, with the nucleus showing higher constraint than genes from either the cytoplasm or the mitochondria. Among metabolic genes, the centrality of an enzyme in the metabolic network is significantly correlated with K_a_/K_s_. In contrast to yeasts, gene expression magnitude does not appear to be the primary predictor of selective constraint in these organisms.

**Conclusions:**

Our results imply that the relationship between selective constraint and enzyme centrality is complex: the strength of selective constraint acting on mammalian genes is quite variable and does not appear to exclusively follow patterns seen in other organisms.

## Background

The rate and manner of evolutionary change has long been a matter of keen interest to biologists [1]. Kimura provided theoretical underpinnings to molecular evolution by relating rates of sequence substitution, population parameters and mutation rates [2,3]. Thus, Kimura's neutral theory [4] predicts that mutations having no fitness effect will become fixed in a population at a rate equal to the mutation rate. Such neutral mutations therefore provide a standard for measuring the action of natural selection: regions changing more slowly than neutral ones are inferred to be experiencing purifying selection (e.g., selective constraint), those changing more rapidly, adaptive evolution. While the relative contributions of genetic drift, adaptive evolution and purifying selection to population differentiation are still debated, [5], there is general agreement that the patterns of selection vary both across species as well as among genes in the same species [6].

Regarding interspecific variation, Lynch and Conery [7] argue that much of the variation in genome structure and content between species can be attributed to differences in their effective population sizes (N_e_). Small effective population sizes limit the efficiency of purifying selection and allow the occasional fixation of mildly deleterious mutations. While some cross-taxa surveys have reported patterns consistent with this hypothesis [8-10], others have found that if one allows for reasonably frequent directional selection there is only a weak relationship between N_e _and selective constraint [11-13].

The second type of variation in selective constraint, that between genetic loci in the same population, has also been studied [14-16]. In particular, considerable effort has gone into identifying factors that predict the selection acting on a particular gene. One critical variable is expression level: mammalian genes expressed in many tissues show stronger selective constraints than do those expressed in only a few tissues [17]. Likewise, in yeast, a high expression level is the primary predictor of strong purifying selection acting on a gene [18], likely because the selective cost of protein misfolding is especially large for highly translated proteins [16].

This association is also in keeping with Wagner's theoretical analyses showing that gene expression is selectively costly in yeast [19]. However, as he notes, the fitness cost of mis-expression is likely to be very different in multicellular organisms [19].

The influence of other factors on selective constraint is also debated, with the evidence primarily coming from studies in yeast [18,20-26]. The topic is confounded by the intercorrelation of many of these predictors [18]. Thus, some researchers report a significant correlation between the fitness cost of gene knockouts and those genes' selective constraint [21,23], while others have questioned this association [20,24]. There is similar debate regarding whether the position of a gene or protein in an interaction network influences selective constraint.

Recall that in these networks genes or proteins are nodes; relationships, such as protein interactions or shared metabolites, are represented as edges between nodes. Researchers have studied the association between selective constraint and measures such as node degree (the number of edges for a given node) and betweenness centrality [a more global statistic measuring the number of shortest paths passing through a node; [27-29]]. Significant associations between node importance and selective constraint have been found in regulatory [30], protein interaction [22], coexpression [31], and metabolic networks [32-34]. However, at least for protein interaction networks, this association seems to be at best quite weak [22,25,26].

**Figure 1:**
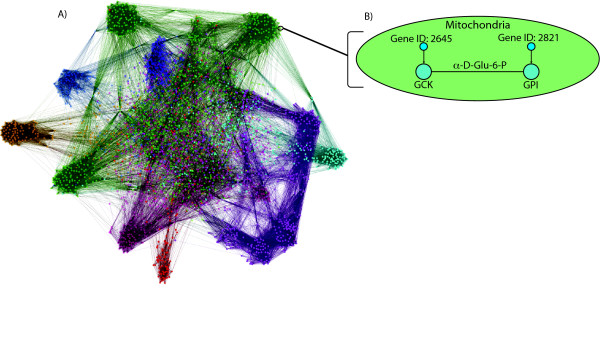
**The human metabolic network**. **A) **The full reaction network used in this analysis. Colors correspond to the compartment in which each reaction occurs. The network was visualized with Gephi 0.7 [63] using the Force-Atlas layout algorithm. **B) **Schematic view of network construction. Reactions that share a metabolite are joined by edges. Each reaction in **A **may also be associated with one or more genes; it is these genes for which we calculate the selective constraint.

Here we explore to what degree these patterns of constraint extend to mammals. Given the difference in lifestyle and effective population size between humans and yeast, we hypothesized that mammals would have evolved in a manner similar to *Drosophila *[34], where there is a significant association between enzyme centrality and evolutionary constraint. We asked whether a gene's position in the human metabolic network (Figure 1) predicts the strength of the purifying selection acting on it. Some previous analyses have calculated the protein divergence between two species, using their common divergence to control for the mutation rate [26]. However, only sampling two sequences offers somewhat limited resolution in the estimation of selective constraint. Here we follow Greenberg, Stockwell and Clark [34] by estimating the selective constraint acting on each human enzyme by comparing it to its orthologs from seven other eutherian genomes (chimpanzee, macaque, mouse, rat, horse, dog and cow). We find that genes encoding metabolic proteins evolve significantly more slowly than other genes. Among those metabolic genes, the encoded protein's cellular compartment is predictive of selective constraint. We also find a weak, though statistically significant, negative correlation between the betweenness of an enzyme in the metabolic network and constraint.

## Results

### Orthology identification

**Figure 2:**
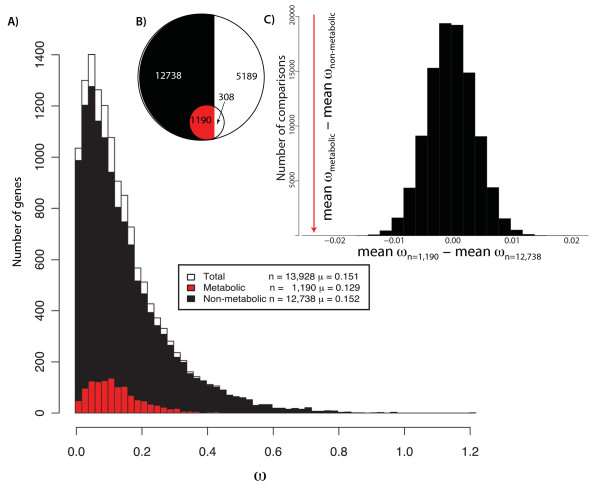
**Human metabolic genes are under greater selective constraint than other orthologous genes**. **A) **The distribution ω for metabolic genes (red, n_m _= 1,190), non-metabolic genes (black, n_o _= 12,738), and the total set of orthologs (white, n_t _= n_m_+n_o_= 13,922). The *x*-axis gives ω: the ratio of non-synonymous to synonymous substitutions per site (i.e., our proxy for selective constraint, see main text). The *y*-axis is the number of genes with a given ω value for each gene set. We can reject the hypothesis that the median of metabolic genes is not significantly smaller than the median of non-metabolic genes (*P *= 0.035; Mann-Whitney U-test). **B) **The white portion of the circle graph shows the relative proportions of genes for which we cannot identify orthologs for the metabolic and non-metabolic genes (red and black, respectively). These proportions are significantly different (χ^2 ^= 11.98, *P *< 0.001). **C) **After taking samples of size *n = *1,190 and *n = *12,738 and calculating the difference in mean ω, 100,000 times, we found no case in which the absolute difference in mean values was as high as the observed difference in mean ω for metabolic and non-metabolic genes (*P *< 10^-5^).

To infer selective constraints for the set of annotated human genes, we identified their orthologs in seven other mammalian genomes using an approach that combines sequence similarity and gene order information (*Methods*). We found 19,416 human genes with at least one ortholog in these genomes. Among those genes, we identified 13,928 sets of orthologs with between 6 and 8 members. Of the 1,496 genes annotated by Duarte et al. [35] as belonging to the metabolic network, 1,190 are in this ortholog set (Figure 2). A greater percentage of genes in the metabolic network fell into our set of orthologs than did genes from the genome at large (χ^2 ^= 47.9; *P *< 0.001; Figure 2B).

### Metabolic and nonmetabolic genes differ in selective constraint

The ratio of nonsynonymous to synonymous substitutions (K_a_/K_s_: hereafter ω) for each set of orthologous genes was estimated by maximum likelihood using PAML 4.2 [Figure 2A; [36]]. This ratio can be interpreted as a measure of selective constraint: values near 0 indicate strong purifying selection, while values greater than 1.0 suggest directional selection.

We hypothesized that metabolic genes would also be under stronger selective constraint than the average non-metabolic gene, so we performed three statistical tests of this hypothesis. First, using a Mann-Whitney U-test (Wilcoxon two-sample test), we rejected the null hypothesis that the median ω among metabolic genes is no smaller than that of non-metabolic genes (i.e., a one-tailed test, *P *= 0.035; Figure 2). Next, we performed a similar test for unequal *mean *ω values between the two groups. Given that neither distribution in Figure 2A appears normal, we adopted a bootstrapping approach, drawing 1,000,000 samples of size *n *= 1,190 from the set of non-metabolic genes (*n *= 12,738) and calculating these samples' means. In no case was the mean value of ω from the bootstrapped samples as small as the observed mean value for metabolic genes (ω_metabolic _= 0.1292, *P *< 10^-6^). We also drew 100,000 samples of sizes *n *= 1,190 and of *n *= 12,738 and calculated the difference in their means. The absolute differences in the mean values was never as large as that observed between the metabolic and non-metabolic genes (*P *< 10^-5^; Figure 2C).

Finally, we performed a more general analysis of the distributions of ω in the two gene sets. To do so, we first fit eight common distributions, the normal, gamma, exponential, Cauchy, log-normal, logistic, Weibull, and extreme value distributions, to the overall set of ω values. We then assessed the quality of the fit of each distribution to the data by analyzing the linear correlation between the ranked data and a Q-Q plot (Table 1; see *Methods*). Out of the eight distributions, three, the Weibull, gamma and exponential provide a visually good fit to the ω values (Additional file 1, Figure S1). For these three distributions, we compared a null model where all genes shared the same distribution parameters to an alternative where the metabolic and non-metabolic genes were allowed to have distinct parameter values for that same distribution. Using a likelihood ratio test, we found that we could reject the null model of identical distributions of ω for the metabolic and non-metabolic genes for all three distributions (*P *< 10^-6^; chi-square distribution; Table 1). Collectively, these three analyses allow us to firmly conclude that metabolic genes are under greater selective constraint than are arbitrary orthologous genes from these genomes.

**Table 1:** Log-likelihoods of a linear fit between all ω values and each of 8 common distributions, with likelihood ratio tests for the differences in distributions calculated for the 3 best distributional fits

Distribution	**Pearson's r**^**a**^	**k**^**b**^	**LRT**^**c**^	df	***P*-value**^**d**^
Weibull	.999	2	186.39	2	<10^-6^
Gamma	.999	2	201.67	2	<10^-6^
Exponential	.998	1	28.29	1	<10^-6^
Logistic	.924	2	-^e^	-	-
Normal	.923	2	-	-	-
Extreme Value	.903	3	-	-	-
Log-Normal	.854	2	-	-	-
Cauchy	.163	2	-	-	-

a. Pearson's *r *is the linear correlation of the data to the quartiles, based on the maximum likelihood inferred parameters for each family of distributions.

b. k is the number of free parameters in the distribution.

c. Likelihood ratio test: LRT = 2 * (log-likelihood of metabolic ω values + log-likelihood of non-metabolic ω values) - (log-likelihood for all ω values).

d. Distributed χ^2^.

e. Likelihood ratio test only performed for the best distributional fits.

### Cellular compartments differ in the selective constraint acting on their enzymes

**Figure 3:**
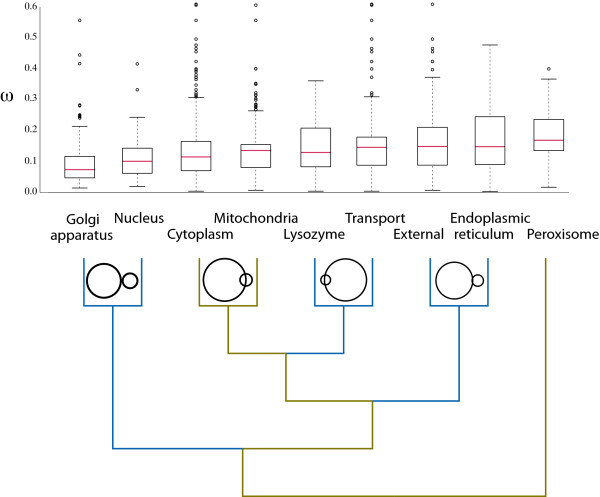
**Hierarchical clustering of cellular compartments based on selective constraint**. Genes are split into nine groups based on their subcellular location (see main text). The median box plots show the distribution of ω values for each compartment - all data, including outliers, are used in the analysis. Using the mean ω values, we created a phenogram using the UPGMA algorithm (branch lengths are arbitrary). Each branch is colored gold if a Mann-Whitney U-test found that the distributions were significantly different at *P *≤ 0.01, and blue otherwise. For example, the cytoplasm and mitochondria distributions are significantly different (*P *= 0.01), but the lysozyme and transport groups show no significant difference (*P *= 0.087). However, when the group formed by the cytoplasm and mitochondria is compared to that formed by the lysozyme and transporters, there is a statistically significant difference (*P *< 0.001). The 4 Venn diagrams show the proportional degree of overlap in genes among groups (sizes are not comparable across nodes in the tree). In none of these cases is any one set of reactions a superset of the other set of reactions.

We next investigated whether an enzyme's tolerance for amino acid substitutions depends on its subcellular localization. This analysis is somewhat less straightforward than it might appear both because some reactions (and hence their enzymes) occur in multiple compartments and because some reactions have multiple isoenzymes. As a result, different cellular compartments can contain the same enzyme. However, the set of overlapping enzymes is in general small and thus unlikely to weaken the power of our analysis significantly (Figure 3). For clarity, we defined proteins involved in transport reactions to be their own distinct category: such reactions have their reactants and products in different compartments.

The mean value of ω varies from 0.0935 in the nucleus to 0.1735 in the peroxisome. To determine if the differences in ω values are significant across compartments, we first clustered the compartments by mean [UPGMA; [37]]. The resulting three groups, in order of increasing ω, are: the Golgi apparatus and the nucleus, all other compartments except the peroxisome, and finally the peroxisome (Figure 3). We tested for significant pairwise differences between compartments in ω using a Mann-Whitney U-test (Figure 3) at a significance level of α = 0.01 (to account for the inherent multiple testing issues). The tests were conducted in a nested fashion, such that groups for which we could not reject the hypothesis of equal values of ω were compared to their nearest neighbors (c.f., the tree in Figure 3). This procedure allowed us to make seven comparisons, rather than the 56 possible pairwise comparisons. We find that the distributions clustered with low ω values (the nucleus and Golgi apparatus) are statistically indistinguishable (*P = *0.047). Those in the large intermediate cluster can be split among groups that are statistically indistinguishable including lysozyme and transport compartments (*P = *0.087), endoplasmic reticulum and external reactions (*P = *0.205), and the cytosol and mitochondrial compartments, which are both statistically distinct from each other (*P *< 0.01). The peroxisome is also statistically distinct from the remaining compartments (*P *< 0.01).

### Network construction

We next explored the role of metabolic network structure in influencing selective constraint, using the metabolic network of Duarte et al. [35]. This network includes information on reaction compartment and directionality that were used to create a semi-directed metabolic network where reactions are nodes. Two nodes are connected by an edge if they share a metabolite. Note that because metabolites are compartment-specific, edges do not connect reactions in differing compartments. Edges are also disallowed if the two reactions in question are irreversible and the interconnecting metabolite serves as a substrate in both reactions or a product in both. The resulting network has 298,004 edges and 3,741 nodes, of which 2,264 have at least one associated gene (Figure 1).

### Removal of currency metabolites

One of the implicit steps in preprocessing metabolic networks is removing currency metabolites, such as water and ATP that participate in numerous reactions. Failing to remove such metabolites prior to analysis can lead to an overestimation of connectedness between reactions.

**Figure 4:**
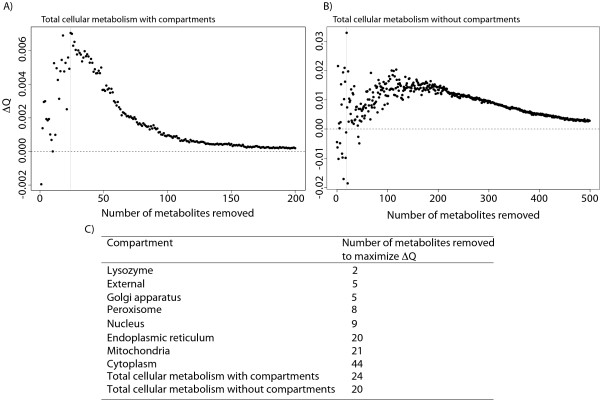
**Exclusion of currency metabolites by maximizing effective modularity**. Effective modularity (ΔQ; *y*-axis) is maximized by iteratively removing common metabolites (*x*-axis). The dashed horizontal line indicates the line corresponding to the ΔQ based on 1000 randomizations of the full network (ΔQ = 0). The vertical line corresponds to the number of metabolites excluded when the network modularity is maximized. **A) **When compartmental designations are included for each metabolite the ΔQ for the network is maximized after the 24 most frequently occurring metabolites are removed. **B) **When compartmental designations are not included for each metabolite the ΔQ for the graph is maximized after the 20 most frequently occurring metabolites are removed. **C) **Results of modularity maximization for the individual cellular compartments.

Rather than introducing an arbitrary cutoff to define currency metabolites, we sought to use to the structure of the network itself to identify them. Other authors have defined and systematically removed currency metabolites from their networks based on their knowledge of the metabolic system [38]. Unfortunately the definition of currency metabolites is not consistent in the literature. Therefore, the network statistic we chose to identify currency metabolites is modularity. Newman [39] defines a measure of optimal modularity, *Q*, as the quality of the subdivision of a network (measured as the fraction of vertices within clustered subdivisions minus the expected fraction of vertices with the same subdivisions in a randomly drawn graph) [40]. Huss and Holme [38] introduce *ΔQ*, which is *Q *for the empirical network minus the average *Q *of a number of random networks. As we remove increasingly less common metabolites, the *ΔQ *of most cellular components has a well-defined maxima (i.e., what modular structure was present in the network is eventually lost as more and more metabolites are removed). Interestingly, when we either consider the network as a whole or the reactions of the cytoplasm alone, the resulting analysis does not present such a well-defined maximal *ΔQ *(Figure 4; Additional file 1, Figure S3), and we propose two reasons for this discrepancy. First, the large number of reactions means that removing certain metabolites (such as H^+^, responsible for half the edges in the network) dramatically changes the network topology, yielding instability in the modularity measurements (see *Methods*). Second, many of the reactions in the cytoplasm are transporters. Because such transport reactions link distinct modules (i.e., compartments) in the network, it is expected that would they behave suboptimally in a modularity analysis.

### Correlations between graph properties and ω

**Figure 5:**
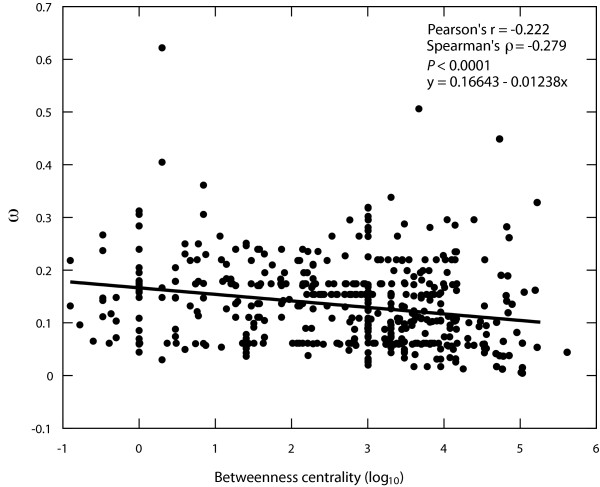
**A negative association of betweenness centrality and selective constraint**. The log_10 _transformed betweenness centrality for each reaction (*x-*axis) is plotted against the estimated selective constraint (ω) for its associated genes. The correlation between these two variables is negative and weak (Pearson's *r *= -0.222, Spearman's *r *= -0.279), but highly significant (*P *< 10^-4^).

We investigated the relationship between two measures of network topology and the selective constraint on the genes associated with network reactions. The measures of reaction importance were the node degree and the betweenness centrality. Interestingly, there is a weak, but statistically significant correlation of betweenness centrality and ω (Figure 5: Spearman's *r *= -0.279, *P *< 10^-4^), but no significant correlation between node degree and ω (Spearman's *r *= -0.029, *P *= 0.075). The network with currency metabolites included shows no relationship between network position and ω (Spearman's *r*_*degree *_= -0.03, *P *= 0.118, *r*_*betweenness *_*= *-0.01, *P = *0.587).

There could be several sources of error associated with such an analysis of network structure and selective constraint. One obvious one is the compartment-by-compartment differences in average selective constraint already described. To explore the role of compartmentalization on this association, we examined the relationships between centrality and ω on a per-compartment basis (Table 2), finding that four compartments had statistically significant association between degree and ω and three had significant associations of betweenness and ω. Oddly, we found a significantly positive association between these variables in the lysozyme.

**Table 2:** Correlations between ω and the graph properties (degree and betweenness) for each compartment, including the number of reactions and edges in each compartment

Compartment	***r***_**degree**_**/ω**^**a**^	***r***_**betweenness**_**/ω**^**b**^	# of reactions	# of edges
Nucleus	0.231	0.024	149	969
Endoplasmic reticulum	0.112	0.045	301	5706
External	-0.093	0.082	986	10279
Golgi apparatus	-0.130	0.103	343	1502
Cytoplasm	-0.168**	-0.193**	2095	196319
Mitochondria	-0.312**	0.043	594	23501
Lysozyme	-0.213*	0.294**	216	7440
Peroxisome	-0.331*	-0.455**	175	1662

a. Spearman's *r *rank correlation of degree and **ω**.

b. Spearman's *r *rank correlation of betweenness-centrality and **ω**.

* *P *< 0.05

** *P *< 0.001

### Positive selection among the metabolic genes cannot explain the associations seen

We found 52 sets of orthologous metabolic genes that showed evidence for positive selection, spread across all cellular compartments (ranging from 0.7% of mitochondrial genes to 6.4% of cytoplasmic ones; see *Methods*). Excluding these genes did not alter our compartment specific estimates of ω, the correlations between network statistics and ω or the significance of the differences in ω between compartments (data not shown).

### There is a weak relationship between gene expression and selective constraint

Using 4,105 genes in both our sample and the HUGE Index [41] we found a weak statistical relationship between ω and maximum expression level (*r = -*0.081; *P *< 10^-6^). For metabolic genes this correlation is somewhat stronger (*r = *-0.089; *P *= 0.029). This relationship, however, is weaker than the relationship we find between network position and ω in metabolic genes, implying that expression may not the dominant predictor of selective constraint in mammals in the same way it is in yeast [18].

## Discussion

Our conclusions that gene function, expression, cellular localization and network position influence selective constraint will individually come as little surprise to researchers. This is especially true of our conclusion that purifying selection acts more strongly on metabolic genes than on genes from the genome at large: function is a known correlate of rate of evolution [42-44].

While we find a significant correlation between reaction centrality (betweenness) and selective constraint in the metabolic network, this result comes with several important caveats. First, although it is reasonable to interpret K_a_/K_s _as the level of selective constraint a gene experiences, in fact, this statistic represents an average evolutionary rate: in particular, two genes with the same fraction of amino acid substitutions forbidden by natural selection might have differing values of K_a_/K_s _if one gene had undergone more adaptive amino acid substitutions. We have partly controlled for this effect by omitting orthologs with evidence of positive selection, but it is not currently possible to completely remove this effect. Another caveat is that the association of betweenness-centrality and (apparent) constraint disappears when the currency metabolites are included. It is also worth noting that node degree on its own is not predictive of constraint in mammals, similar to the lack of association between these variables seen in *E. coli *[26]. We suggest one useful message to take from this result is that the relationship that exists between selective constraint and betweenness centrality is dependent on the manner in which the network is constructed. Special care has been taken in justifying the removal of currency metabolites across networks, however different removal strategies produce different associations of centrality and constraint (*Methods*).

From a more general perspective, it is also important to recall that networks are only computational abstractions of a biological reality. To speak of an association of betweenness and selection is therefore actually to suggest that betweenness, a measurable quality, also represents an underlying biological feature. In this work, we have not directly demonstrated such a biological association. Likewise, there is a difference between the metabolic network associations seen here and those in protein interaction networks. In protein interaction networks, the pairwise binding of proteins is directly mediated by sequences, and natural selection can act to maintain complementary sequences in two interacting proteins [45]. In metabolic networks, the relationship is more tenuous; one assumes that central reactions are required for proper function of the metabolic network and hence enzymes catalyzing such reactions will be under greater constraint. Even if this argument holds, the constraint is on function and not specifically on sequence. If an enzyme can maintain this function using differing sequences, there might be no necessary association of sequence constraint and centrality.

When we break the metabolic network down by compartment, we do find associations between network centrality (degree or betweenness) and constraint in some, but not all, compartments (Table 2). One lesson from these complex results is that although it is intuitive to consider the relationship between metabolic network structure and selective constraint at a global level, differences in constraint among compartments may confound global analyses. Likewise, the variation in constraint among these compartment raises interesting questions: it is unclear why enzymes from the Golgi apparatus and nucleus should be more highly conserved than those from the central group of compartments (Figure 3). Strikingly, enzymes implicated in external reactions fall within this central group, and are not distinguished by having a uniquely fast or slow rate of substitution. This result contrasts the findings by Liao et al. [46] and Julenius and Pedersend [47] that the intra-/extra-cellular localization of a protein is highly predictive of its ω. However, note that these authors considered all genes in a given compartment, as opposed to the strictly metabolic ones analyzed here.

One potential explanation for these differences in constraint between compartments is that those compartments have different tolerances for misfolded proteins. Protein misfolding appears to have a significant fitness cost in yeast [16], and it is not unreasonable to hypothesize that the spatial organization of the nucleus [48] might induce a particularly high cost for misfolded proteins. However, one observation that speaks against this hypothesis is the weak association of constraint and expression. Our results thus suggest that although gene expression in some manner constrains mammalian protein evolution, it is less effective at doing so in mammals than in yeast.

## Conclusions

In general, we find that although the position of a mammalian gene's product in the metabolic network and its expression level are both associated with that gene's evolutionary constraint, neither factor is determinative. Thus, unlike yeast, the forces that determine the selective constraint on mammalian protein-coding genes are likely both to be complex and to vary between genes.

## Methods

### Orthology identification

Our method for orthology identification first detects homologous genes using sequence similarity and then uses gene order to resolve orthology [orthology and paralogy are reviewed in [49]]. Specifically, we first conduct a pairwise homology search among all genes in *G1 *and *G2 *using GenomeHistory [50]. GenomeHistory hits were filtered to exclude those with E-values greater than 10^-10 ^(comparisons to chimpanzee and macaque) or 10^-9 ^(all other comparisons) and amino acid sequence identity less than 50% (chimpanzee and macaque) or 45% (all others). Cases where two homologous genes are immediate neighbors on a chromosome (e.g., tandem duplicates) are treated as a single locus. An initial ortholog pair *A *and *B *is inferred if three criteria are met:

• *A *is from *G1 *and *B *is from *G2*.

• The only homology of *A *in *G2 *is *B *and the only homology of *B *in *G1 *is *A*.

• The synonymous divergence between *A *and *B *is less than a threshold (K_s _< 0.5 for the human-chimpanzee and human-macaque comparisons and <0.75 in all other cases).

Many genes in *G1 *and *G2 *will have multiple homologs and hence not fall into a one-to-one relationship. Instead, we use this smaller set of one-to-one relationships to detect further orthologs. First, define *C *as the immediate (left or right) neighboring gene of *A *and *D *as the neighbor of *B*. If *C *and *D *are homologs, even if they also show homology to other genes, they are defined as orthologs. Importantly, now that *C *and *D *are identified as orthologs, their other homology relationships are deleted. We repeat the procedure for identifying one-to-one pairs, no longer using criterion 3. The entire process is repeated until no further orthologs are identified [9].

### Sequence alignment and quality control

We created a data pipeline using Bioperl 1.6 [51]. The initial inputs were the set of gene orthologs determined above: we found 19,416 ortholog sets for the 8 eutherian mammal species. This set is made up of protein-coding genes with no clear evidence of tandem duplication, since duplication and subsequent functional specialization can alter measured selective constraints [52]. Human genes with orthologs in fewer than 5 other mammals were excluded from analysis.

Given a set of orthologous genes from humans and at least five other mammals, the corresponding amino acid sequences were aligned using MUSCLE v3.6 with default parameters [53]. We next performed several filtering steps to assure alignment quality. First, we required that all possible pairs of sequences in each alignment have pairwise percent identity (PID) of ≥40%. If any pair of sequences had a PID < 40%, then the sequence with the lowest PID to a consensus sequence was removed. The remaining sequences were then realigned and their PID rechecked. Next, we removed gap columns from the finished alignments. In cases where this resulted in fewer than 50 aligned amino acid columns, the sequences with the lowest PID to the consensus sequence was removed and the original sequences were realigned. This was done iteratively as long as there were still more than 5 sequences to align. The result of these filtering steps was the 13,928 multiple sequence alignments used in the remainder of our analyses.

### Estimation of selective constraint

Using the above amino acid alignments, we inferred codon-preserving nucleotide alignments and estimated the ratio of nonsynonymous to synonymous rates (K_a_/K_s _or ω) with the codeml package in PAML 4.2 [36]. We assumed the sequences had evolved under previously published mammalian phylogenetic relationships [54,55], namely ((((human, chimpanzee), macaque), (mouse, rat)),((horse, dog), cow)).

PAML model M0 was used to estimate the maximum likelihood value of ω [56]. Recall that the synonymous substitution rate is used as a proxy for the mutation rate: the deficit or surplus of nonsynonymous substitutions relative to this value is then indicative of purifying or diversifying selection.

### Identification of metabolic genes under positive selection

With these same data, we used a site-specific model to look for genes under positive selection. We compared PAML models M1a and M2a, nearly-neutral and positive selection models, respectively [57]. M1a has two ω parameters: ω_0 _< 1 and ω_1 _= 1. M2a has three ω parameters: ω_0 _< 1, ω_1 _= 1, and ω_2 _> 1. A likelihood ratio test was used to determine if M2a was a significantly better fit to the data than M1a, given that model M2a has two more free parameters. Genes that had twice the difference in log-likelihood greater than the critical χ^2 ^value (5.99; *P *< 0.05) were assumed to be under positive selection.

### Gene expression analysis

We collected expression levels for 4,105 genes by querying Affimetrix microarray data in the HUman Gene Expression Index [HUGE Index; [41]].We then determined the maximum level at which a gene is expressed in the 19 tissues, comprising 59 experiments in the HUGE Index database.

### Distributional fits

We tested whether the metabolic and non-metabolic ortholog sets had differing values of ω using a nonparametric Mann-Whitney U-test [58] as implemented in R. Differences in ω between cellular compartments were also analyzed with this test.

As discussed in the *Results*, because the data in question were visually skewed, we sought to confirm the results of the Mann-Whitney test of differences in ω between metabolic and nonmetabolic genes in two ways. First, we bootstrapped samples of size *n *= 1,190 (the number of metabolic orthologs in our dataset) from the set of non-metabolic ω values and compared the sample means to the actual mean ω of the metabolic genes. Second, to compare not only the means of the two sets of genes but also their variability, we fit several probability density functions to these data using the MASS library in R [59]. The distributional parameters were estimated by maximum likelihood: numerical optimization was carried out using Nelder-Mead or Broyden-Fletcher-Goldfarb-Shanno methods for single and multi-parameter distributions, respectively [59]. We used these estimations to calculate the Pearson's correlation coefficient for Q-Q plots of quantile values versus observed frequency (Additional file 1, Figure S1). The result of this analysis was a list of distributional families ranked in terms of the best fit of each distribution to the data (Table 1).

We used a likelihood ratio test [LRT; [58]] to compare the distributions of metabolic and non-metabolic ω values. The null model requires the set of ω values from both metabolic and non-metabolic genes to be drawn from a single probability distribution. The alternative model allows the metabolic and non-metabolic genes to have differing values of the distribution parameters (while still following the same distribution function). As a result, the alternative model has twice as many free parameters (2*p*) as does the null model, allowing us to compare the difference in log likelihood between the two models to a χ^2 ^distribution with *p *degrees of freedom.

### Network properties

Modularity, degree, and betweenness estimates for the metabolic networks were calculated using the igraph library [60] in R. Modularity was estimated using the Clauset et al. algorithm for detecting community structure [61]. Betweenness was calculated using the Brandes algorithm [27,28,62].

### Removal of currency metabolites

As discussed in the *Results*, and following Huss and Holme [38], we defined currency metabolites as metabolites whose removal increased the effective modularity (*ΔQ*) of the network in question. Modularity is a measure of the degree to which nodes fit into distinct and connected subunits [39]. Effective modularity is the difference between the maximum modularity of the graph and the average maximum modularity of a number of random graphs [38]. Removing currency metabolites increases modularity, since it removes interconnections between distinct subgraphs, while removing non-currency metabolites decreases modularity by isolating reactions from their subgraph modules.

To calculate *ΔQ *we compared modularity (*Q*) [39] of the graph to the average *Q *of 1000 randomly rewired graphs. The *Q *for the graph minus the average *Q *of these 1000 random graphs is *ΔQ *[38]. We thus ordered the metabolites by the frequency with which they formed edges, removed the most frequent metabolite and calculated *ΔQ*. We then reordered the metabolites and repeated this procedure until any further removal of metabolites only decreased *ΔQ*.

We optimized *ΔQ *for three different types of networks. First, we used a noncompartmentalized network (i.e., we removed compartmental metabolite designations so that ATP in the cytoplasm is treated equivalently to ATP in the mitochondria, Figure 4A). We next considered a network where we retained compartmental metabolite designations (where a specific metabolite may be removed from one cellular component, but not another) and optimized the global compartmentalized network (Figure 4B; Additional file 2, Table S1). Finally, we optimized the modularity in each cellular compartment individually (Additional file 1, Figure S3). Because the compartmentalized network offered both improved biological intuition and better performance in our modularity analysis, it was used for all of the analyses presented above. We note, however, this noncompartmentalized network shows a weaker association of betweenness and ω than does the compartmentalized one (*r*_*betweenness *_*= *-0.07, *P *< 10^-4^).

## Authors' contributions

CMH and GCC developed the concept of the experiments and wrote the manuscript. CMH performed the experiments and analyzed the data. Both authors read and approved the final manuscript.

## Supplementary Material

Additional file 1 **Supplemental figures**. **Figure S1** - Q-Q plots are for 8 common distributions. Weibull, gamma, exponential, logistic, normal, extreme value, log-normal and Cauchy. The X-Y line is y = x. The *x*-axis plots the theoretical quartiles for a statistical population from one of the 8 distributions, while the *y*-axis plots the data. Values that lie on the line y = x are a good fit between the theoretical distribution and data. The Weibull, gamma, and exponential distributions provide close visual fits to the data (see Table 1 for the correlations). **Figure S2** - Ortholog identification. Homologous genes within and between genomes are first identified based on a lack of within-genome paralogs in both genomes. We then identify each pair of genes that are immediate neighbors of a pair of orthologs and are also homologous. Because these genes have other homologs in the other genome, they were not part of the initial ortholog list. We now define them as orthologs, and at the same time, remove any orphan genes that no longer show homology to genes in the other genome not already in orthologous pairs. Using the new pairs, we repeat the process until no further orthologs are located. **Figure S3** - Maximum effective modularity for each compartment and for the total cellular metabolic network. Effective modularity (ΔQ) on the *y*-axis is maximized for each of the subcellular compartments, including organelles, external reactions, and the cytoplasm by iteratively removing each of the most common metabolites (the number on the *x*-axis). The dashed horizontal line indicates the line corresponding to the ΔQ based on 1000 random iterations (ΔQ = 0). The vertical line corresponds to the points where the graph is maximized. Click here for file

Additional file 2 **Supplementary Table S1 - **Compartment specific currency metabolites removed from total network. Click here for file

## References

[B1] Simpson GG Tempo and mode in evolution 1944 New York: Columbia University Press

[B2] Kimura M Evolutionary rate at the molecular level Nature 1968 217 624 626 10.1038/217624a0 5637732

[B3] Kimura M The rate of molecular evolution considered from the standpoint of population genetics Proc Natl Acad Sci USA 1969 63 1181 1188 10.1073/pnas.63.4.1181 5260917 PMC223447

[B4] Kimura M The neutral theory of molecular evolution 1983 Cambridge: Cambridge University Press

[B5] Nei M Selectionism and neutralism in molecular evolution Mol Biol Evol 2005 22 2318 2342 10.1093/molbev/msi242 16120807 PMC1513187

[B6] Cooper GM Brudno M Stone EA Dubchak I Batzoglou S Sidow A Characterization of evolutionary rates and constraints in three mammalian genomes Genome Res 2004 14 539 548 10.1101/gr.2034704 15059994 PMC383297

[B7] Lynch M Conery JS The origins of genome complexity Science 2003 302 1401 1404 10.1126/science.1089370 14631042

[B8] Ellegren H A selection model of molecular evolution incorporating the effective population size Evolution 2009 63 301 305 10.1111/j.1558-5646.2008.00560.x 19215289

[B9] Conant G Neutral evolution on mammalian protein surfaces Trends Gen 2009 25 377 381 10.1016/j.tig.2009.07.004 19716195

[B10] Slotte T Foxe JP Hazzouri KM Wright SI Genome-wide evidence for efficient positive and purifying selection in *Capsella grandiflora*, a plant species with a large effective population size Mol Biol Evol 2010 27 1813 1821 10.1093/molbev/msq062 20194429

[B11] Fay JC Wyckoff GJ Wu CI Testing the neutral theory of molecular evolution with genomic data from *Drosophila* Nature 2002 415 1024 1026 10.1038/4151024a 11875569

[B12] Charlesworth J Eyre-Walker A The other side of the nearly neutral theory, evidence of slightly advantageous back-mutations Proc Natl Acad Sci USA 2007 104 16992 16997 10.1073/pnas.0705456104 17940029 PMC2040392

[B13] Bachtrog D Similar rates of protein adaptation in *Drosophila miranda *and *D. melanogaster*, two species with different current effective population sizes BMC Evol Biol 2008 8 334 10.1186/1471-2148-8-334 19091130 PMC2633301

[B14] Fitch WM Margoliash E Construction of phylogenetic trees Science 1967 155 279 284 10.1126/science.155.3760.279 5334057

[B15] Yang Z Maximum-likelihood estimation of phylogeny from DNA sequences when substitution rates differ over sites Mol Biol Evol 1993 10 1396 1401 8277861 10.1093/oxfordjournals.molbev.a040082

[B16] Drummond DA Bloom JD Adami C Wilke CO Arnold FH Why highly expressed proteins evolve slowly Proc Natl Acad Sci USA 2005 102 14338 14343 10.1073/pnas.0504070102 16176987 PMC1242296

[B17] Duret L Mouchiroud D Determinants of substitution rates in mammalian genes: expression pattern affects selection intensity but not mutation rate Mol Biol Evol 2000 17 68 85 10666707 10.1093/oxfordjournals.molbev.a026239

[B18] Drummond DA Raval A Wilke CO A single determinant dominates the rate of yeast protein evolution Mol Biol Evol 2006 23 327 337 10.1093/molbev/msj038 16237209

[B19] Wagner A Energy constraints on the evolution of gene expression Mol Biol Evol 2005 22 1365 1374 10.1093/molbev/msi126 15758206

[B20] Hurst LD Smith NG Do essential genes evolve slowly? Curr Biol 1999 9 474 450 10.1016/S0960-9822(99)80334-0 10421576

[B21] Hirsh AE Fraser HB Protein dispensability and rate of evolution Nature 2001 411 1046 1049 10.1038/35082561 11429604

[B22] Fraser HB Hirsh AE Steinmetz LM Scharfe C Feldman MW Evolutionary rate in the protein interaction network Science 2002 296 750 752 10.1126/science.1068696 11976460

[B23] Jordan IK Rogozin IB Wolf YI Koonin EV Essential genes are more evolutionarily conserved than are nonessential genes in bacteria Genome Res 2002 12 962 968 12045149 10.1101/gr.87702 PMC1383730

[B24] Pál C Papp B Hurst LD Rate of evolution and gene dispensability Nature 2003 421 496 497 10.1038/421496b 12556881

[B25] Jordan IK Wolf YI Koonin EV No simple dependence between protein evolution rate and the number of protein-protein interactions: only the most prolific interactors tend to evolve slowly BMC Evol Biol 2003 3 1 10.1186/1471-2148-3-1 12515583 PMC140311

[B26] Hahn MW Conant GC Wagner A Molecular evolution in large genetic networks: Connectivity does not equal constraint J Mol Evol 2004 58 203 211 10.1007/s00239-003-2544-0 15042341

[B27] Freeman LC A set of measures of centrality based on betweenness Sociometry 1977 40 35 41 10.2307/3033543

[B28] Brandes U A faster algorithm for betweenness centrality J of Math Sociol 2001 25 163 177 10.1080/0022250X.2001.9990249

[B29] Liu WC Lin WH Davis AJ Jordan F Yang HT Hwang MJ A network perspective on the topological importance of enzymes and their phylogenetic conservation BMC Bioinformatics 2007 8 121 10.1186/1471-2105-8-121 17425808 PMC1955749

[B30] Jovelin R Phillips CP Evolutionary rates and centrality in the yeast gene regulatory network Genome Biol 2009 10 R35 10.1186/gb-2009-10-4-r35 19358738 PMC2688926

[B31] Jordan IK Marino-Ramirez L Wolf YI Koonin EV Conservation and coevolution in the scale-free human gene coexpression network Mol Biol Evol 2004 21 2058 2070 10.1093/molbev/msh222 15282333

[B32] Vitkup D Kharchenko P Wagner A Influence of metabolic network structure and function on enzyme evolution Genome Biol 2006 7 R39 10.1186/gb-2006-7-5-r39 16684370 PMC1779518

[B33] Wagner A Evolutionary constraints permeate large metabolic networks BMC Evol Biol 2009 9 231 10.1186/1471-2148-9-231 19747381 PMC2753571

[B34] Greenberg AJ Stockwell SR Clark AG Evolutionary constraint and adaptation in the metabolic network of *Drosophila* Mol Biol Evol 2008 25 2537 2546 10.1093/molbev/msn205 18799713 PMC2721553

[B35] Duarte NC Becker SA Jamshidi N Thiele I Mo ML Vo TD Srivas R Palsson BØ Global reconstruction of the human metabolic network based on genomic and bibliomic data Proc Natl Acad Sci USA 2007 104 1777 1782 10.1073/pnas.0610772104 17267599 PMC1794290

[B36] Yang Z PAML 4: Phylogenetic analysis by maximum likelihood Mol Biol Evol 2007 24 1586 1591 10.1093/molbev/msm088 17483113

[B37] Sneath PHA Sokal RR Numerical Taxonomy 1973 San Francisco: W. H. Freeman & Co

[B38] Huss M Holme P Currency and commodity metabolites: their identification and relation to the modularity of metabolic networks IET Systems Biology 2007 1 280 285 10.1049/iet-syb:20060077 17907676

[B39] Newman MEJ Modularity and community structure in networks Proc Natl Acad Sci USA 2006 103 8577 8582 10.1073/pnas.0601602103 16723398 PMC1482622

[B40] Newman MEJ Girvan M Finding and evaluating community structure in networks Physical Review E 2004 69 026113 10.1103/PhysRevE.69.026113 14995526

[B41] Haverty P Weng Z Best N Auerbach K Hsiao LL Jensen R Gullans S HugeIndex: a database with visualization tools for high-density oligonucleotide array data from normal human tissues Nucleic Acids Res 2002 30 214 217 10.1093/nar/30.1.214 11752297 PMC99144

[B42] De S Lopez-Bigas N Teichmann SA Patterns of evolutionary constraints on genes in humans BMC Evol Biol 2008 8 275 10.1186/1471-2148-8-275 18840274 PMC2587479

[B43] Lopez-Bigas N De S Teichmann SA Functional protein divergence in the evolution of *Homo sapiens* Genome Biol 2008 9 R33 10.1186/gb-2008-9-2-r33 18279504 PMC2374701

[B44] Tuller T Kupiec M Ruppin E Co-evolutionary networks of genes and cellular processes across fungal species Genome Biol 2009 10 R48 10.1186/gb-2009-10-5-r48 19416514 PMC2718514

[B45] Bloom JD C A Apparent dependence of protein evolutionary rate on number of interactions is linked to biases in protein-protein interactions data sets BMC Evol Biol 2003 3 21 10.1186/1471-2148-3-21 14525624 PMC270031

[B46] Liao B Weng M Zhang J Impact of extracellularity on the evolutionary rate of mammalian proteins Genome Biol Evol 2010 2 39 43 10.1093/gbe/evp058 20333223 PMC2839354

[B47] Julenius K Pedersen AG Protein evolution is faster outside the cell Mol Biol Evol 2006 22 2039 2048 10.1093/molbev/msl081 16891379

[B48] Fraser P Bickmore W Nuclear organization of the genome and the potential for gene regulation Nature 2007 447 413 417 10.1038/nature05916 17522674

[B49] Koonin E Orthologs, paralogs, and evolutionary genomics Annual Review of Genetics 2005 39 309 338 10.1146/annurev.genet.39.073003.114725 16285863

[B50] Conant GC Wagner A GenomeHistory: A software tool and its application to fully sequenced genomes Nucleic Acids Res 2002 30 3378 3386 10.1093/nar/gkf449 12140322 PMC137074

[B51] Stajich JE Block D Boulez K Brenner SE Chervitz SA Dagdigian C Fuellen G Gilbert JGR Korf I Lapp H The Bioperl Toolkit: Perl modules for the life sciences Genome Res 2002 12 1611 1618 10.1101/gr.361602 12368254 PMC187536

[B52] Kondrashov FA Rogozin IB Wolf YI Koonin EV Selection in the evolution of gene duplications Genome Biol 2002 3 research0008 10.1186/gb-2002-3-2-research0008 11864370 PMC65685

[B53] Edgar R MUSCLE: multiple sequence alignment with high accuracy and high throughput Nucleic Acids Res 2004 32 1792 1797 10.1093/nar/gkh340 15034147 PMC390337

[B54] Murphy WJ Pevzner PA O'Brien SJ Mammalian phylogenomics comes of age Trends Gen 2004 20 631 639 10.1016/j.tig.2004.09.005 15522459

[B55] Nishihara H Hasegawa M Okada N Pegasoferae, an unexpected mammalian clade revealed by tracking ancient retroposon insertions Proc Natl Acad Sci USA 2006 103 9929 9934 10.1073/pnas.0603797103 16785431 PMC1479866

[B56] Yang Z Nielsen R Goldman N Pedersen A-MK Codon-substitution models for heterogeneous selection pressure at amino acid sites Genetics 2000 155 431 449 10790415 10.1093/genetics/155.1.431 PMC1461088

[B57] Wong W Yang Z Goldman N Nielsen R Accuracy and power of statistical methods for detecting adaptive evolution in protein coding sequences and for identifying positively selected sites Genetics 2005 168 1041 1051 10.1534/genetics.104.031153 PMC1448811 15514074

[B58] Sokal R Rohlf FJ Biometry 2000 3 New York: W. H. Freeman and Company

[B59] Venables WN Ripley BD Modern Applied Statistics with S 2002 Fourth New York: Springer

[B60] Csárdi G Nepusz T The igraph software package for complex network research InterJournal, Complex Systems 2006 1695

[B61] Clauset A Newman MEJ Moore C Finding community structure in very large networks Phys Rev E 2004 70 066111 10.1103/PhysRevE.70.066111 15697438

[B62] Freeman LC Centrality in social networks I: conceptual clarificaiton Social Networks 1979 1 215 239 10.1016/0378-8733(78)90021-7

[B63] Bastian M Heymann S Jacomy M Gephi: an open source software for exploring and manipulating networks International AAAI Conference on Weblogs and Social Media 2009

