# Supplementary material for: Expression level, cellular compartment and metabolic network position all influence the average selective constraint on mammalian enzymes
Source: BMC Evol Biol. 2011 Apr 6;11:89. doi: 10.1186/1471-2148-11-89 (PMC3082228; doi:10.1186/1471-2148-11-89)
Supplement: Additional file 1 — Supplemental figures. Figure S1 - Q-Q plots are for 8 common distributions. Weibull, gamma, exponential, logistic, normal, extreme value, log-normal and Cauchy. The X-Y line is y = x. The x-axis plots the theoretical quartiles for a statistical population from one of the 8 distributions, while the y-axis plots the data. Values that lie on the line y = x are a good fit between the theoretical distribution and data. The Weibull, gamma, and exponential distributions provide close visual fits to the data (see Table 1 for the correlations). Figure S2 - Ortholog identification. Homologous genes within and between genomes are first identified based on a lack of within-genome paralogs in both genomes. We then identify each pair of genes that are immediate neighbors of a pair of orthologs and are also homologous. Because these genes have other homologs in the other genome, they were not part of the initial ortholog list. We now define them as orthologs, and at the same time, remove any orphan genes that no longer show homology to genes in the other genome not already in orthologous pairs. Using the new pairs, we repeat the process until no further orthologs are located. Figure S3 - Maximum effective modularity for each compartment and for the total cellular metabolic network. Effective modularity (ΔQ) on the y-axis is maximized for each of the subcellular compartments, including organelles, external reactions, and the cytoplasm by iteratively removing each of the most common metabolites (the number on the x-axis). The dashed horizontal line indicates the line corresponding to the ΔQ based on 1000 random iterations (ΔQ = 0). The vertical line corresponds to the points where the graph is maximized. [file 1471-2148-11-89-S1.PDF]

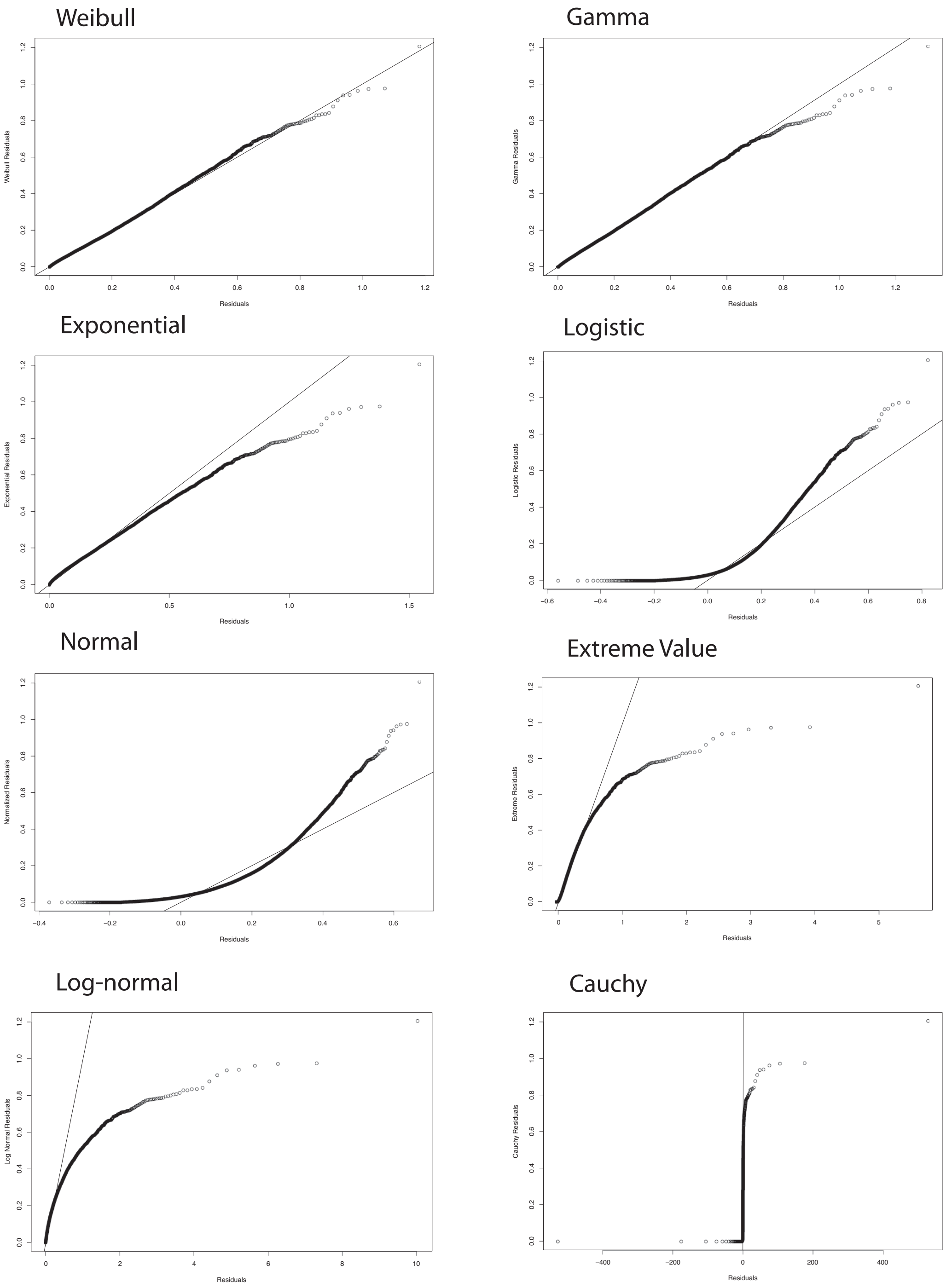


**Supplementary Figure S1 - Q-Q plots are for 8 common distributions.** Weibull, gamma, exponential, logistic, normal, extreme value, log-normal and Cauchy. The X-Y line is y = x. The x-axis plots the theoretical quartiles for a statistical population from one of the 8 distributions, while the y-axis plots the data. Values that lie on the line y = x are a good fit between the theoretical distribution and data. The Weibull, gamma, and exponential distributions provide close visual fits to the data (see Table 1 for the correlations).

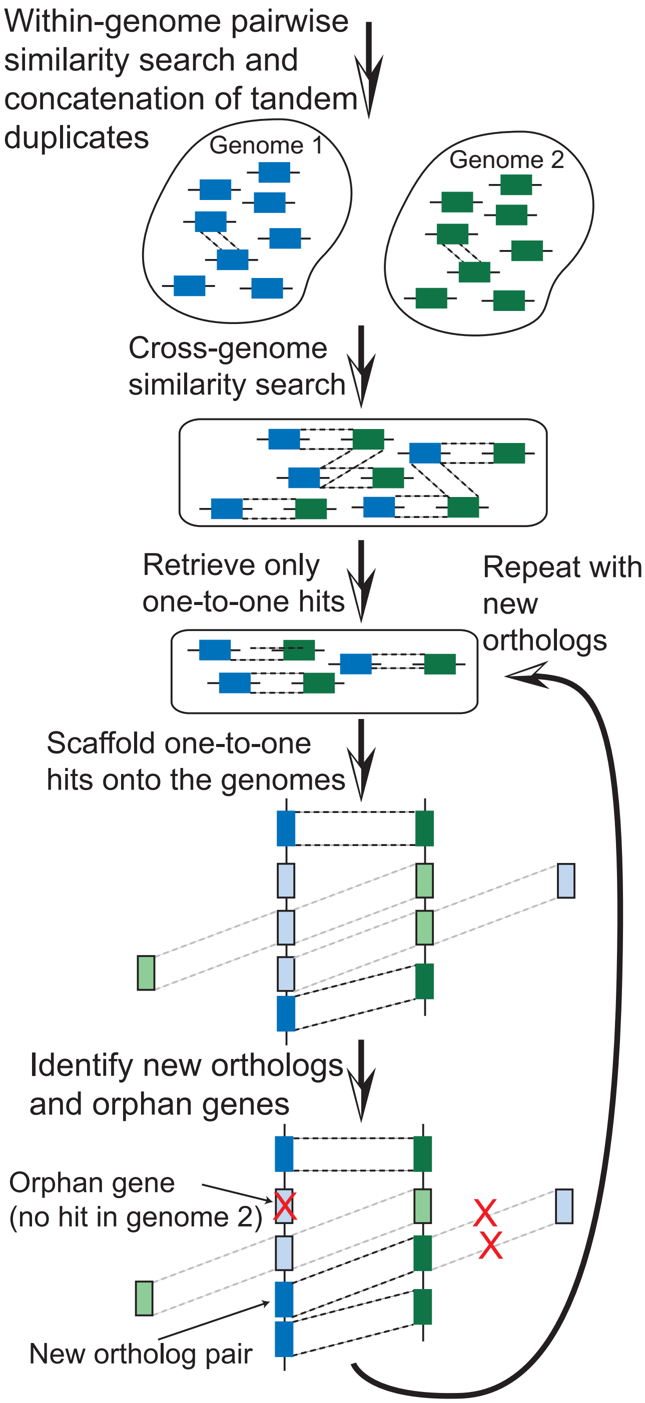


**Supplementary Figure S2 - Ortholog identification.** Homologous genes within and between genomes are first identified based on a lack of within-genome paralogs in both genomes. We then identify and pair of genes that are immediate neighbors of a pair of orthologs and are also homologous. Because these genes have other homologs in the other genome, they were not part of the initial ortholog list. We now define them as orthologs, and, at the same time, remove any orphan genes that no longer show homology to genes in the other genome not already in orthologous pairs. Using the new pairs, we repeat the process until no further orthologs are located.

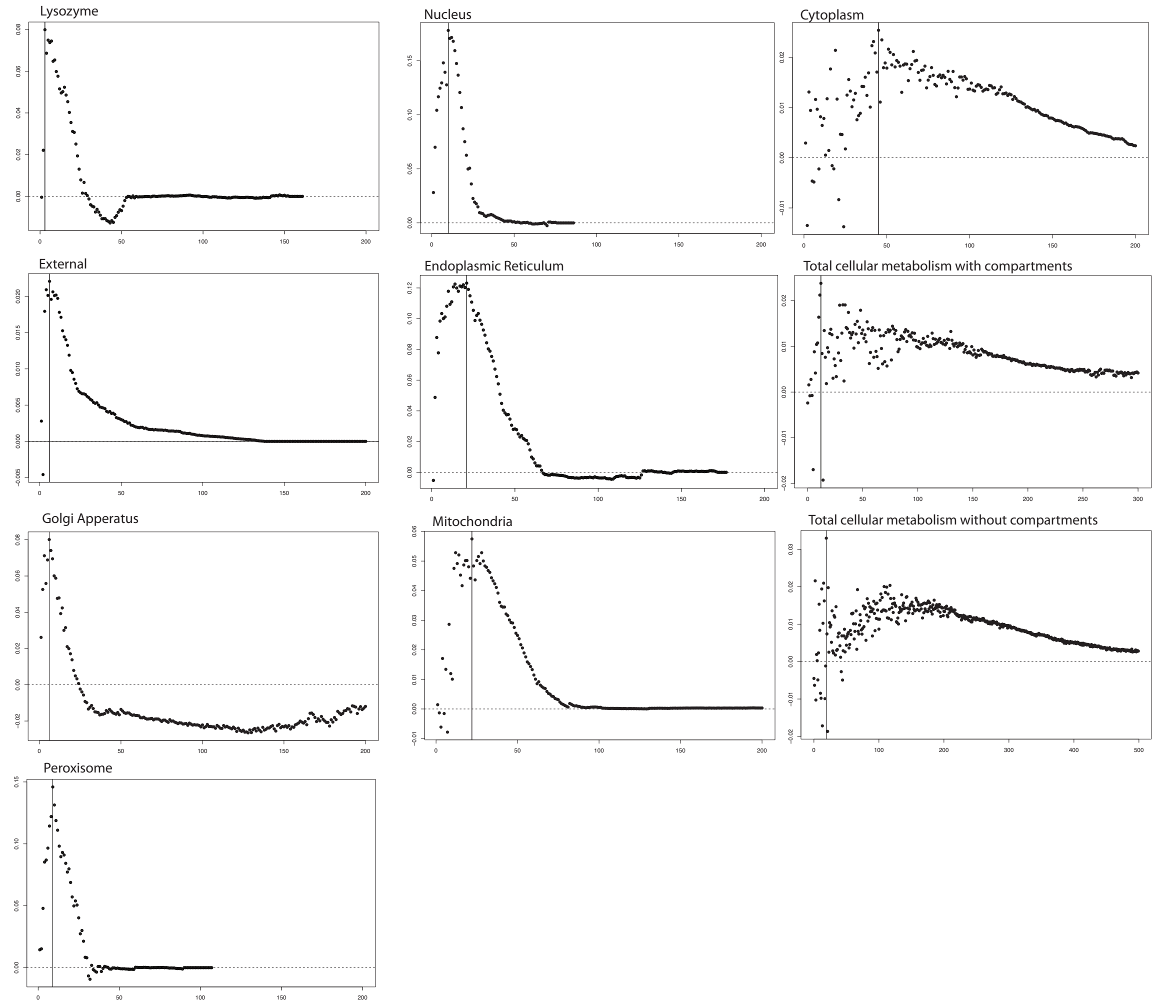


**Supplementary Figure S3 - Maximum effective modularity for each compartment and for the total cellular metabolic network.** Effective modularity (ΔQ) on the y-axis is maximized for each of the subcellular compartments, including organelles, external reactions, and the cytoplasm by iteratively removing each of the most common metabolites (the number on the x-axis). The dashed horizontal line indicates the line corresponding to the ΔQ based on 1000 random iterations (ΔQ = 0). The vertical line corresponds to the points where the graph is maximized.
