# Supplementary material for: Expression level, cellular compartment and metabolic network position all influence the average selective constraint on mammalian enzymes
Source: BMC Evol Biol. 2011 Apr 6;11:89. doi: 10.1186/1471-2148-11-89 (PMC3082228; doi:10.1186/1471-2148-11-89)
Supplement: Additional file 2 — Supplementary Table S1 - Compartment specific currency metabolites removed from total network. [file 1471-2148-11-89-S2.DOC]

**Supplemental Table S1. Compartment specific currency metabolites removed from total graph.**

|  | Compartment | | | | | | | |
| --- | --- | --- | --- | --- | --- | --- | --- | --- |
| Removed Metabolite | Cytoplasm | Mitochondria | Nucleus | Endoplasmic  Reticulum | Golgi apparatus | External | Lysozyme | Peroxisome |
| M_h^a^ | Yes | Yes | No | Yes | Yes | Yes | Yes | No |
| M_h2o | Yes | Yes | No | Yes | No | No | Yes | No |
| M_atp | Yes | No | No | No | No | No | No | No |
| M_na1 | Yes | No | No | No | No | No | No | No |
| M_adp | Yes | Yes | No | No | No | No | No | No |
| M_coa | Yes | Yes | No | No | No | No | No | No |
| M_pi | Yes | No | No | No | No | No | No | No |
| M_nad | Yes | No | No | No | No | No | No | No |
| M_amp | Yes | No | No | No | No | No | No | No |
| M_nadp | Yes | No | No | No | No | No | No | No |
| M_ppi | Yes | No | No | No | No | No | No | No |
| M_fad | No | Yes | No | No | No | No | No | No |
| M_o2 | Yes | No | No | No | No | No | No | No |
| M_glu_DASH_L | Yes | No | No | No | No | No | No | No |

a. Metabolite nomenclature following Duarte et al. [1]

References:

1. Duarte NC, Becker SA, Jamshidi N, Thiele I, Mo ML, Vo TD, Srivas R, Palsson Bò: **Global reconstruction of the human metabolic network based on genomic and bibliomic data.** *Proc Natl Acad Sci U S A* 2007, **104:**1777-1782.
